# A Decade in Banking Ewing Sarcoma: A Report from the Children’s Oncology Group

**DOI:** 10.3389/fonc.2013.00057

**Published:** 2013-03-20

**Authors:** Scott C. Borinstein, Natalie Beeler, John J. Block, Richard Gorlick, Patrick Grohar, Paul Jedlicka, Mark Krailo, Carol Morris, Sharon Phillips, Gene P. Siegal, Elizabeth R. Lawlor, Stephen L. Lessnick, COG Ewing Sarcoma Biology Committee

**Affiliations:** ^1^Division of Pediatric Hematology/Oncology, Department of Pediatrics, Vanderbilt UniversityNashville, TN, USA; ^2^Children’s Oncology Group, Biopathology CenterColumbus, OH, USA; ^3^Department of Radiology and Radiological Sciences, Vanderbilt UniversityNashville, TN, USA; ^4^Division of Pediatric Hematology/Oncology, Department of Pediatrics, Montefiore Medical CenterNew York, NY, USA; ^5^Department of Pathology, Children’s Hospital Colorado, Anschutz Medical Campus, University of Colorado DenverAurora, CO, USA; ^6^Department of Statistics, Children’s Oncology GroupMonrovia, CA, USA; ^7^Department of Orthopedic Surgery, Memorial Sloan-Kettering Cancer Center, Weill Cornell Medical CollegeNew York, NY, USA; ^8^Department of Biostatistics, Vanderbilt UniversityNashville, TN, USA; ^9^Department of Pathology, UAB and the UAB Comprehensive Cancer Center, University of AlabamaBirmingham, AL, USA; ^10^Division of Pediatric Hematology/Oncology, Department of Pediatrics, University of MichiganArbor, MI, USA; ^11^Division of Pediatric Hematology/Oncology, Department of Oncological Sciences, The Center for Children’s Cancer Research, Huntsman Cancer InstituteSalt Lake City, UT, USA

**Keywords:** Ewing sarcoma, tumor banking, biopsy, interventional radiology, bone tumor, adolescent and young adult

## Abstract

Outcomes for patients with metastatic and recurrent Ewing sarcoma remain poor and a better understanding of the biology of this malignancy is critical to the development of prognostic biomarkers and novel therapies. Therefore, the Children’s Oncology Group (COG) has created tissue banking protocols designed to collect high quality, clinically annotated, tumor specimens that can be distributed to researchers to perform basic science and correlative investigation. Data from the COG Ewing sarcoma tissue banking protocols AEWS02B1 and its successor study AEWS07B1 were reviewed in this study. Six-hundred and thirty five patients were enrolled on AEWS02B1 and 396 patients have had tissue submitted to AEWS07B1. The average age of participation was 13.2 years. About 86% were less than 19 years old and only 6% were greater than 21 years of age at diagnosis. When compared to SEER data, approximately 18% of all cases and only 8% of all patients >20 years old diagnosed with Ewing sarcoma annually in the United States have had tumor banked. The majority of participants submitted formalin fixed, paraffin embedded, primary tumor and blood samples. In total, fresh frozen tissue was submitted for only 29% of cases. Only seven metastatic tumor samples have been collected. Although the COG has been successful in collecting tumor samples from patients newly diagnosed with Ewing sarcoma, fresh frozen tumor specimens from primary and metastatic disease are critically needed, especially from young adult patients, in order to conduct high quality basic science and translational research investigation with a goal of developing better treatments.

## Introduction

Ewing sarcoma is a solid tumor of bone and soft tissue that primarily afflicts adolescents and young adults (Pizzo and Poplack, 2006; Karski et al., 2013). Ewing sarcoma can arise in any bone of the body, although the most common sites of disease include the pelvis, ribs, and long-bones of the extremities. Approximately 25% of patients have metastatic disease at diagnosis, most often found in the lungs, bone, and bone marrow. The treatment of Ewing sarcoma relies on a multidisciplinary approach, coupling highly intensive chemotherapy with surgery, and/or radiotherapy for control of the primary site of disease and metastatic lesions (Arndt and Crist, 1999). Patients with overt metastases have a <30% disease-free survival, while those with disease localized to a single site generally have a >70% disease-free survival at 5 years following diagnosis (Rodriguez-Galindo et al., 2003; Womer et al., 2012). Furthermore, patients with metastatic disease have not experienced improvements in outcomes in over 30 years (Grier et al., 2003).

Ewing sarcomas are characterized genetically by the presence of recurrent chromosomal translocations. In over 85% of cases the translocation occurs between chromosomes 11 and 22 resulting in the creation of a pathognomonic chimeric fusion gene, *EWSR1/FLI1* that encodes the EWS/FLI protein (Delattre et al., 1992). Alternative chromosomal translocations between members of the *EWSR1* and *ETS* gene families, such as *t*(21;22), *EWSR1/ERG*, have been identified in the majority of cases that do not have a classic *EWSR1/FLI1* fusion (Khoury, 2005). Since its discovery 20 years ago, great insights into the biology of the EWS/FLI protein in the initiation and progression of Ewing sarcoma have been gained, but these discoveries remain to be translated into novel therapeutic strategies. A critical issue has been that the EWS/FLI protein has not been effectively targeted by current agents in the clinical setting. Thus, many investigators have sought to better understand the underlying biology of Ewing sarcoma with the goal of identifying new therapeutic strategies that might be more efficacious and less toxic than current chemotherapeutic approaches.

An important requirement for understanding the biology of Ewing sarcoma is access to primary patient materials. Such materials include not only tumor tissue, but also specimens of bone marrow, blood, serum, and genomic DNA (both tumor and germ line). Shortly after the Children’s Oncology Group (COG) was formed by the merger of four North American-centered pediatric cancer groups [Pediatric Oncology Group (POG), Children’s Cancer Group (CCG), Intergroup Rhabdomyosarcoma Study Group (IRSG), and National Wilms Tumor Study Group (NWTS)], a commitment to obtaining and banking specimens from patients with pediatric cancers was conceived. Collection of samples was made on prior therapeutic studies, but these were protocol-specific and not organized with a larger strategy in place.

The strategic Ewing sarcoma banking effort was initiated with the development of COG protocol AEWS02B1, “A Groupwide Biology and Banking Study for Ewing sarcoma,” which opened on January 21, 2003, and closed on July 7, 2008. This study was replaced by COG protocol AEWS07B1, “A Children’s Oncology Group Protocol for Collecting and Banking Ewing Sarcoma Specimens,” which opened for patient entry on February 4, 2008, and remains open at the time of this report. The goal of these tumor banking studies is to collect high quality tumor specimens with associated demographic and clinical data that can be distributed to researchers to perform basic science and correlative investigation that will improve the diagnosis, staging, risk-stratification, and treatment of Ewing sarcoma.

As the COG-based Ewing sarcoma banking effort approaches its tenth anniversary, we recognize the opportunity to review this effort and to provide an overview of the resources that are available to the research community. In this report we will highlight some of the successes of these efforts, some challenges, and some issues that have yet to be resolved. Finally, we provide information on the process for sample requests and approval.

## Design and Aims of the COG Ewing Sarcoma Tumor Banking Studies

### AEWS02B1

The goal of the AEWS02B1 tumor banking study was to develop a mechanism to collect and distribute tumor specimens to investigators conducting basic science and translational research on Ewing sarcoma. In addition, several important biologic aims were incorporated into the study design, including determination of (1) the prognostic significance of translocation subtype in Ewing sarcoma; (2) the role of minimal residual disease (MRD) detection in bone marrow specimens by RT-PCR determination of EWS/ETS fusion genes; and (3) whether serum levels of IGF1 and IGFBP-3 are associated with clinical outcome. Additional exploratory aims included analysis of gene expression profiles to identify novel molecular targets for treatment in Ewing sarcoma, establishment of a bank of xenografts in SCID mice, and exploration of clinical proteomics as a resource for investigations of altered signaling molecules in the pathogenesis of Ewing sarcoma.

All participating centers had appropriate IRB approval and informed consent was and/or assent was obtained prior to patient enrollment. For each enrolled patient, institutions were required to provide either a formalin fixed, paraffin embedded (FFPE) tissue block or 10–20 FFPE unstained slides in addition to 1–3 thick 50 μm sections. Whole blood and serum specimens were also required. It was strongly recommended that additional specimens be submitted including a bone marrow aspirate (2–4 ml in EDTA), FFPE tissue blocks or slides obtained at the time of surgical resection and recurrence, and frozen primary tumor tissue frozen in OCT media sent on dry ice.

### AEWS07B1

The AEWS07B1 tumor banking study was designed to continue to collect, bank, and store Ewing sarcoma tumor specimens. Unlike AEWS02B1, there were no specific biologic aims included in the study. Patients were required, at the time of diagnosis or recurrence, to submit a FFPE tissue block or 20 unstained FFPE slides, whole blood and serum, and a pre-treatment bone marrow aspirate (supplied as 2–4 ml in EDTA containing tubes). It was also strongly recommended that additional frozen tumor samples preserved in either OCT media, RNA Later (Qiagen, Valencia, CA, USA), or flash frozen in liquid nitrogen be submitted. For both AEWS02B1 and AEWS07B1 protocols, each was approved by an Institutional Review Board and informed consent was obtained from all participants.

## Results

### Patient demographics and study accrual

**Figure 1:**
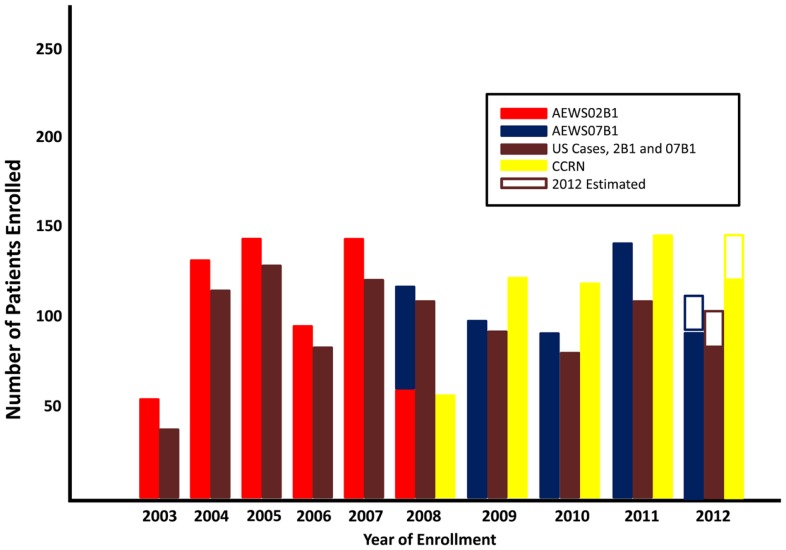
**Study accrual**. Data as of March 21, 2012.

Between 2003 and 2008 a total of 635 patients from 85 institutions were enrolled on AEWS02B1. Thirteen patients were declared ineligible either due to misdiagnosis, errors in the consent process, or inadequate acquisition or shipment of tumor specimens. AEWS07B1 was opened in 2008 and as of October 30, 2012, 470 patients were enrolled from 152 institutions. There has been consistent and steady accrual on the tumor banking studies since AEWS02B1 was opened on January 21, 2003. Fifty-six patients were enrolled in 2003 and this low accrual was likely secondary to institutional administrative delays in opening. Accrual was doubled in 2004 (133 patients) and has remained relatively constant with an average enrollment of 119 patients per year between 2004 and 2011 (Figure 1). There was a slight decrease in accrual from 2008–2010 likely attributed to the fact that there was not an open therapeutic trial for localized Ewing sarcoma patients during this time. The majority of patients enrolled on either banking study were from the United States, as would be expected for a COG study (brown bars, Figure 1).

Starting in 2007, COG opened the Cancer Control Registry Network (CCRN), with a goal of registering all newly diagnosed patients with cancer treated at COG institutions. To determine what proportion of patients with Ewing sarcoma were being captured for AEWS07B1 up to age 21 (the CCRN age limit for enrollment), we compared registration on the banking study with the CCRN database. Since the collection of CCRN data, approximately 80% of patients newly diagnosed with Ewing sarcoma were also enrolled on AEWS07B1 (range 77–92%), reinforcing the hypothesis that younger patients (less than 21 years old) treated at COG institutions are most likely to enroll on a tissue banking study. Surveillance, Epidemiology, and End Result (SEER) data from 2000–2009 estimates 574 new cases of bone and soft tissue Ewing sarcoma each year in the United States and 321 cases under the age of 20 (SEER, 2011). These data suggest that although accrual of patients who were diagnosed and treated at COG affiliated institutions was high (80% of all patients enrolled on the CCRN), only 18% (range 14–23%) of all predicted cases of Ewing sarcoma and 28% (range 22–35%) of patients <20 years old in the United States were enrolled on the banking studies.

**Figure 2:**
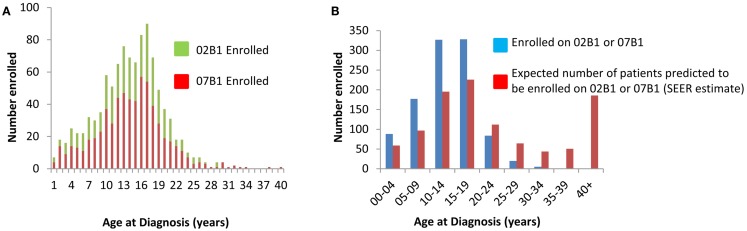
**(A)** Age of patient at time of sample submission. **(B)** Predicted enrollment per SEER estimates on 02B1 and 07B1 enrollment. Data as of March 21, 2012.

Demographic data were collected for all enrolled patients and are summarized in Table 1. The mean age for both studies was approximately 13 years. As shown in Figure 2A, the majority of patients were in their teenage years at diagnosis and relatively few adult tumor samples were banked. Specifically, 86% of patients that banked tumor specimens were less than 19 years old and only 6% were greater than 21 years old at the time of diagnosis. Per SEER estimates, 44% of patients should be older than 20 years, with an expected 191 patients diagnosed annually in the U.S. Thus, only 8% (range 6–10%) of patients >20 years were enrolled on these studies.

**Table 1:** **Patient Demographics^1^**.

	AEWS02B1	AEWS07B1	Total
Sex			
Male	357 (56%)	237 (60%)	594
Female	278 (44%)	159 (40%)	437
Age (years)			
0–4	54	34	88
5–9	108	69	177
10–14	204	123	327
15–19	197	131	328
20–24	52	32	84
25+	20	7	27

*^1^ As of March 21, 2012*.

To further illustrate the bias toward younger patients enrolled on AEWS02B1 and AEWS07B1, the age of enrollment on the COG biology protocols was compared to the predicted age estimate. In this analysis, the number of patients at each age subset enrolled on study was compared to the expected number at each age range extrapolated from SEER estimates. For example, the SEER data expects that 22% of all patients diagnosed with Ewing sarcoma are between the ages of 15–19. Therefore, it would be expected that 226 patients would have enrolled on the tumor banking studies were 15–19 at diagnosis. In actuality, 328 patients ages 15–19 were enrolled on the study, or 32% of the total patient population, suggesting that enrollment is biased toward younger patients. Furthermore, when compared to SEER data from 2003 until 2009, 67% of patients diagnosed with EWS should be less than 20 years old. However, as shown in Figure 2B, 89% of patients were 19 years old or younger and significantly fewer patients over 25 years old were enrolled on study than what would be expected.

### Banked tissue samples

Table 2 lists the inventory of banked tumor specimens from both AEWS02B1 and AEWS07B1. Serum and FFPE tissue were collected from the majority of all participating patients (86 and 85%, respectively). Bone marrow was submitted from 400 patients (38%), and this percentage substantially increased after AEWS07B1 opened. Fresh frozen (including OCT embedded) tissue was banked in only 29% of patients (*n* = 304). Metastatic tumors were even more rarely banked and, to date, tissue from metastatic sites has been submitted for only seven patients (0.7%).

**Table 2:** **Inventory of banked tumor specimens^1^**.

Specimen	AEWS02B1	AEWS07B1	Total
Formalin fixed paraffin embedded primary tissue	524	345	869
Fresh tissue	182	122	304
FFPE metastatic tissue	1	1	2
Fresh metastatic tissue	4	1	5
Serum/plasma	551	336	887
Blood	310	343	653
Bone marrow, fresh	88	312	400
Bone marrow, FFPE	4	2	6
Urine	7	2	9
**Type(s) of biological tissue submitted per patient**			
FFPE + blood	261	29	290
FFPE + blood + BM	54	181	235
FFPE + fresh + blood	125	18	143
FFPE + fresh + blood + BM	24	91	115
BLOOD only	62	6	68
FFPE only	44	15	59
None	24	9	33
Blood + BM	5	26	31
FFPE + fresh	14	5	19
Fresh + blood	13	1	14
FFPE + BM	2	4	6
Fresh + blood + BM	2	4	6
FRESH only	4	1	5
BM only	1	4	5
FFPE + fresh + BM	0	2	2
Fresh + BM	0	0	0
	635	396	1031

*^1^ As of March 21, 2012*.

*Formalin Fixed Paraffin Embedded (FFPE) primary tissue – includes unstained slides, paraffin blocks, scroll*.

*Fresh tissue – includes OCT, snap frozen in liquid nitrogen, RNA later (Qiagen, CA, USA)*.

*Blood – includes Ficolled WBC, serum, and plasma, exclude unstained peripheral blood slides*.

*Bone marrow, Fresh – includes Ficolled WBC or fresh frozen*.

*Bone marrow, FFPE – includes unstained slides*.

*Urine – includes frozen*.

*Not included – Normal tissue, Miscellaneous samples not otherwise specified*.

**Figure 3:**
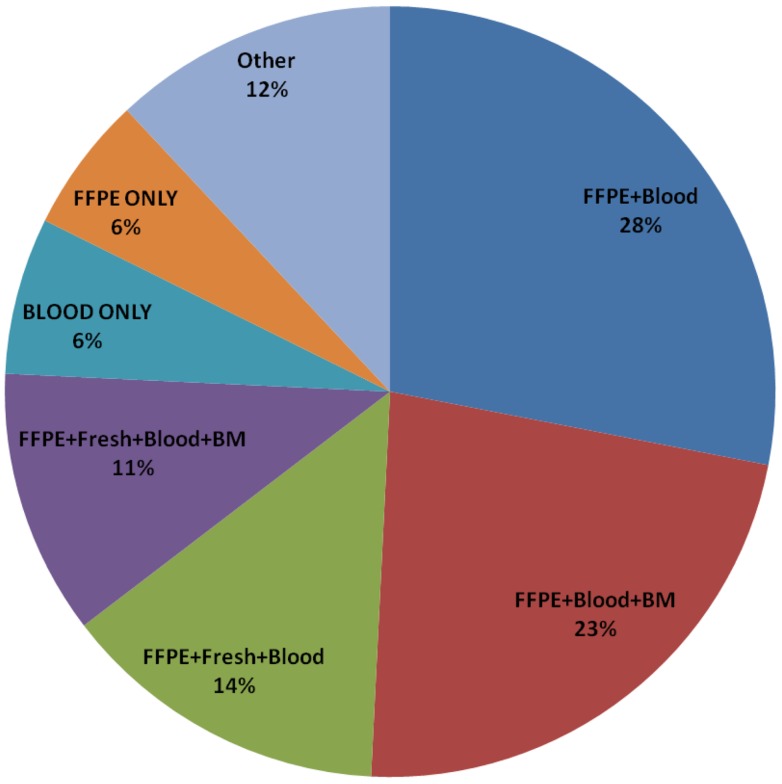
**Distribution of tumor specimens**.

Table 2 and Figure 3 list the combinations of specimens submitted per patient. The most frequent combination submitted by participating institutions was FFPE tissue (block or unstained slides) and blood (*N* = 260, 28%), which were required on both AEWS02B1 and AEWS07B1. Institutions that submitted only stained hematoxylin and eosin stained slides were not counted, resulting in 68 patients only submitting blood samples. The majority of these patients (62 out of 68) were enrolled on AEWS02B1. FFPE, blood, and bone marrow were submitted in 235 patients (23%), but only 115 out of 1031 enrolled patients submitted blood, bone marrow, FFPE, and fresh tissue (11%). About 29% of enrollments included fresh frozen tumor tissue (Table 2).

### Correlative studies using banked specimens and published manuscripts

The purpose of the COG banking studies is to provide a resource of high quality biologic materials that can be used for research investigation. Several defined COG group wide studies were included within the aims of AEWS02B1 whereas AEWS07B1 was designed to be more amenable to individual investigator-initiated research. To evaluate the relative success of these banking studies we evaluated the distribution of samples to investigators and the resulting publications that included data from these studies. Although this was not a systematic approach to identify all research projects that have used Ewing sarcoma tissue bank samples, Table 3 lists a subset of the published manuscripts that have used these samples. These projects have contributed to both basic science discovery and translational biology investigation in addition to providing special collections of tissue samples necessary to answer critical biologic questions. These banked tumor specimens have contributed to the generation of novel scientific information that has resulted in publication in high impact scientific journals (Table 3).

**Table 3:** **Published manuscripts that have used tumor samples collected from AEWS02B1 and AEWS07B1**.

Author (year)	Title	Journal
Douglas et al. (2008)	BMI-1 promotes Ewing sarcoma tumorigenicity independent of CDKN2A repression	Cancer Research
Joo et al. (2009)	Gli1 is a central mediator of EWS/Fli1 signaling in Ewing tumors	PLoS ONE
Bennani-Baiti et al. (2010)	Intercohort gene expression co-analysis reveals chemokine receptors as prognostic indicators in Ewing’s sarcoma	Clinical Cancer Research
Dubois et al. (2010)	Flow cytometric detection of Ewing sarcoma cells in peripheral blood and bone marrow	Pediatric Blood and Cancer
Jiang et al. (2010)	CD133 expression in chemo-resistant Ewing sarcoma cells	BMC Cancer
van Doorninck et al. (2010)	Current treatment protocols have eliminated the prognostic advantage of type 1 fusions in Ewing sarcoma: a report from the Children’s Oncology Group	Journal of Clinical Oncology
Borinstein et al. (2011)	Investigation of the insulin-like growth factor-1 signaling pathway in localized Ewing sarcoma: a report from the Children’s Oncology Group	Cancer
Cooper et al. (2011)	Ewing tumors that do not overexpress BMI-1 are a distinct molecular subclass with variant biology: a report from the Children’s Oncology Group	Clinical Cancer Research
Solomon et al. (2011)	Mutational inactivation of STAG2 causes aneuploidy in human cancer	Science
von Levetzow et al. (2011)	Modeling initiation of Ewing sarcoma in human neural crest cells	PLoS ONE
Jahromi et al. (2012)	Molecular inversion probe analysis detects novel copy number alterations in Ewing sarcoma	Cancer Genetics

## Discussion

Since the Ewing sarcoma biology studies were opened and began accruing patients in 2003, blood, tumor, and bone marrow specimens have been banked from over 1000 patients with newly diagnosed Ewing sarcoma. An estimated 18% of all cases of Ewing sarcoma diagnosed in the United States were successfully banked and as a result of these efforts, tumors and associated biologic specimens have been distributed to investigators across North America. The data generated by these studies have in turn generated basic science and translational insights that have advanced our understanding of this malignant neoplasm. Tumor specimens are banked at the Biopathology Center in Columbus, Ohio, and are managed by the Ewing Sarcoma Bone Tumor Committee. The Committee’s goal is to facilitate research focused on Ewing sarcoma that will ultimately have an impact on the care of patients with Ewing sarcoma. Thus, the Committee must balance a number of competing needs. On the one hand, the Committee strives to ensure that all tumor bank specimens are being utilized in a rigorous and scientific manner. On the other hand, the Committee tries to ensure that samples in the tumor bank are available to investigators both now and in the future.

To ensure that these competing needs are met, the Ewing Sarcoma Biology Committee has developed a sample request process that starts with the submission of an investigator-initiated application. The application is reviewed by three Committee members as well as the Chair or Vice-Chair of the Committee. The Committee reviews applications for feasibility, use of resources, scientific quality, and likely impact. A statistical review is also conducted to ensure that studies are adequately powered to address the question proposed. If the initial review is positive, the application is then discussed by the full Committee to ensure alignment of the study with COG goals. Investigators are notified of the review outcome via written response. This process ensures that all specimen requests are reviewed in an objective and transparent manner to balance the needs of the investigator with the mission of COG and the Committee. This strategy has not only resulted in the generation of many high quality publications, but has also ensured a large inventory of tissue specimens for future studies.

However, despite these successes, the COG Ewing sarcoma biology studies have had limitations. First, while there have been great numbers of patients enrolled on the studies, the amount of tumor specimens has lagged, especially with regards to snap-frozen specimens. This has created some difficulties in gathering large numbers of high quality specimens required for some “genome-wide” studies, such as RNA-based transcriptional profiling. One might question why snap-frozen specimens are only “recommended” rather than “required” for enrollment on Ewing sarcoma banking studies. At issue is an attempt to balance ease of patient enrollment (such that enrollment does not become restricted) with the need for sample submission. It is clear that many institutions only obtain limited amounts of primary tumor specimen at initial biopsy. Once tumor material has been used for diagnostic purposes, in many instances, very little remains for banking purposes. At this time, there is no biomarker required for enrollment onto a therapeutic study, and so it is unethical to require additional tumor collection simply to satisfy the interest in expanding the tumor bank. The Ewing Sarcoma Biology Committee is currently studying a number of potential biomarkers that might find use in therapeutic stratification of patients. If such a biomarker is identified, then it would become clinically relevant and appropriate to require sample submission for enrollment onto a therapeutic study.

A second challenge is that neither biology study provides central review or independent molecular confirmation (i.e., FISH or RT-PCR investigation of EWS fusions) of tumor specimens. This information is available only if collected by the submitting institution. Centralized review of all tumor specimens might not only increase accrual (by providing an additional “expert” pathology second opinion for all enrolled patients), but would provide researchers additional confidence that all tumor specimens have a confirmed pathologic diagnosis. This has become of greater concern in recent years with the realization that there are a group of “Ewing-like sarcomas” that phenocopy the *bona fide* disease, but have alternate rare translocations (Sankar and Lessnick, 2011). While these look-alike tumors are likely to constitute only a small portion of enrolled patients, further study will be required to fully understand the scope of this issue. At this time the COG Ewing sarcoma biology Committee does not have the financial resources required to perform central review of all submitted specimens.

A final challenge is that there is a serious need for tumor specimens collected from patients greater than 20 years of age, from metastatic sites, and from patients at relapse. Young adults with Ewing sarcoma have a worse event free survival than younger patients as do patients with metastatic disease at diagnosis (Cotterill et al., 2000; Yock et al., 2006; Leavey et al., 2008; Borinstein et al., 2011). Whether there are specific biologic factors that lead to this worse outcome is currently unknown. Investigation of this age-based outcome discrepancy is warranted, but cannot be fully addressed without a suitable collection of tumor specimens from these cohorts of patients. Therefore, we strongly encourage surgeons, pathologists, medical oncologists, and pediatric oncologists to work together to make tumor banking a priority. Adults are often treated in hospitals that are not COG institutions. However, most academic medical centers are affiliated with pediatric oncology programs that can assist in patient enrollment. These interactions not only will result in increased participation of older patients on Ewing sarcoma tumor banking studies, but will foster cooperation between adult and pediatric providers and will result in better care for our patients. Furthermore, the Sarcoma Alliance for Research through Collaboration (SARC) has recently been awarded a Specialized Program of Research Excellence (SPORE) grant. One of the aims of this project is to collect Ewing sarcoma tumor specimens from adult patients. Given the close relationship between SARC and COG, we are optimistic that this endeavor will result in the acquisition of more Ewing sarcoma tumors from older individuals that can be used for scientific discovery.

In light of these shortcomings, the COG Ewing sarcoma biology committee proposes the following set of recommendations that should result in improved accrual and availability of high quality specimens and to produce a bank of tumors that will be suitable for current molecular analytic techniques and also valuable for future investigation.

### Banking of fresh tissue from pre-treatment surgical biopsy: Recommendations

Fresh viable tumor tissue represents an invaluable source of material for clinically relevant studies in cancer biology. The ideal specimen for obtaining such tissue is from a pre-treatment biopsy, a sample obtained during the confirmation of relapse or metastases, or surgical resection of a tumor. For some solid tumors, such as Wilms tumors, which are resected prior to therapy, banking fresh tissue is relatively feasible. However, for tumors such as Ewing sarcoma, which are often only biopsied, from not easily accessible sites, and then treated prior to resection, banking fresh tissue represents a greater challenge. Nonetheless, it is feasible in many cases, and facilitated by a close working relationship between surgeon or radiologist, pathologist and, if available, a local institutional COG coordinator.

**Figure 4:**
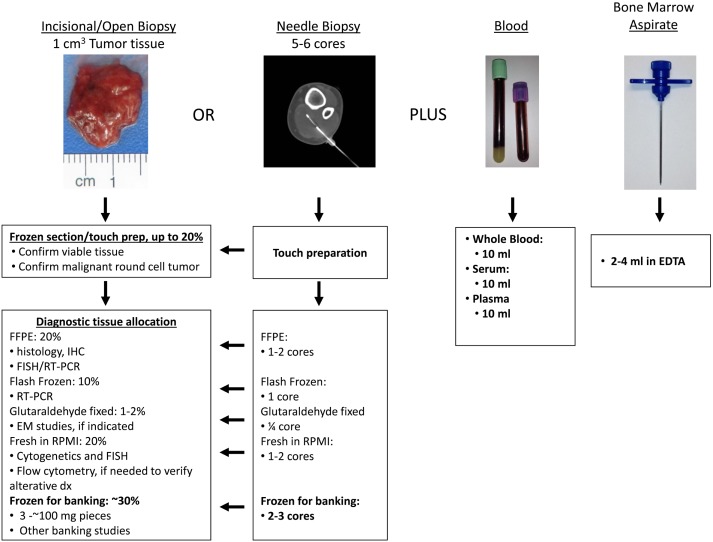
**Optimal procurement of Ewing sarcoma tumor samples**.

A possible protocol for fresh tissue banking is illustrated using a real case in Figure 4. As for all tumor biopsies, the optimal specimen is roughly a cubic centimeter of tumor tissue. After such tissue is obtained by the surgeon, a frozen section or touch preparation is performed by the pathologist. This is usually part of the routine diagnostic protocol in order to verify viable tumor tissue, provide an intraoperative differential diagnosis and guide specimen allocation for optimal diagnostic work-up. Depending on pathologist/institutional preference, if Ewing sarcoma is in the differential diagnosis based on the frozen section or touch preparation, allocation might include any or all of the steps outlined in Figure 4. All cases will include allocation of more viable tumor tissue for permanent histology and immunohistochemical studies. Depending on institutional practice, FFPE tissue can also be used for FISH and RT-PCR molecular diagnostic studies, to support the diagnosis of Ewing sarcoma or an alternative pathologic process. If electron microscopic facilities are available, a small amount of tissue may be preserved in glutaraldehyde for such studies. If facilities for cytogenetic studies are available, fresh tissue may be sent for cytogenetics and FISH. It is probably always prudent to freeze at least a small amount of viable tumor tissue for other potential studies if needed, including molecular diagnostic studies on fresh tissue. Additional fresh tissue may also be saved for possible flow cytometry, should subsequent pathologic examination of FFPE tissue suggest a hematologic malignancy rather than Ewing sarcoma. Once the above allocation has taken place, any remaining tissue may be banked. In the real example illustrated in Figure 4, approximately 30% of fresh biopsy tissue was banked without compromising the clinical diagnosis.

**Figure 5:**
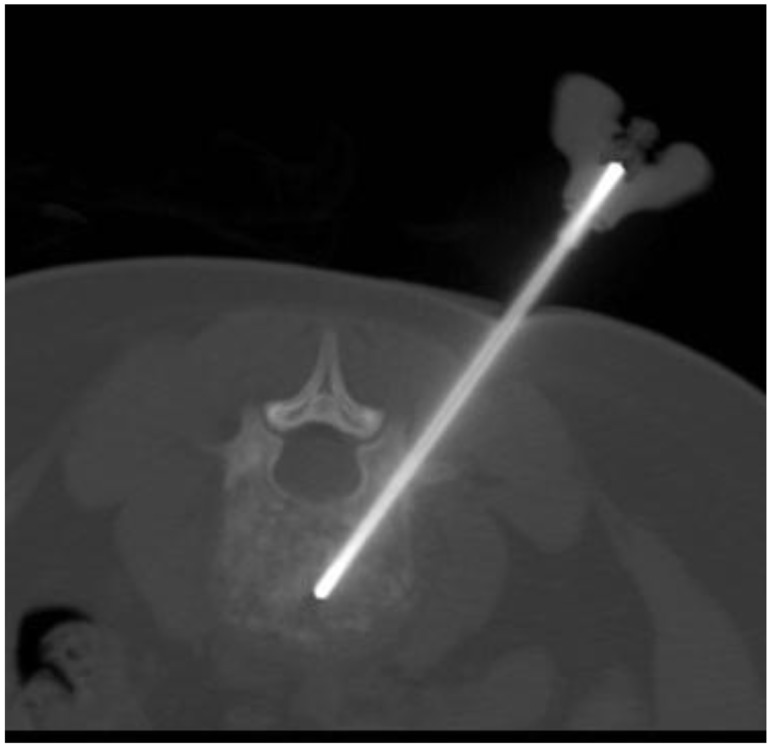
**Interventional biopsy of a bone lesion: this figure depicts a CT image of a vertebral bone biopsy using an 11-gage biopsy needle**. Optimum tumor acquisition is feasible with a smaller number of passes.

With increasing incidence, especially for difficult-to-access tumor sites, needle biopsy, conducted by either the surgeon or by an interventional radiologist, is performed to acquire diagnostic tumor tissue. Given the lack of tissue volume obtained using these procedures, this poses significant impediment for tissue banking studies. However, these percutaneous biopsy procedures may be optimized for the retrieval of adequate tissue and the avoidance of complications which might alter the overall care of the patient. In order to respect compartmental anatomy and plan for the subsequent surgical approach with the necessity for resection of the biopsy tract, image-guided biopsies are often performed in consultation with surgical oncology input (Anderson et al., 1999). Coaxial CT-guided biopsies, as shown in Figure 4, optimize documentation of the needle path utilized, and allow for fine needle aspiration (FNA) samples to be obtained prior to core needle biopsy of non-sclerotic osseous and soft tissue lesions. Given the heterogeneity of some bone and soft tissue cancers, real-time cytopathologist consultation of FNA samples assures that core biopsies obtained from the same coaxial trajectory does represent viable lesional tissue rather than necrotic or non-diagnostic material. In the event that the cytopathologist determines FNA samples are inadequate, redirection prior to core biopsy is warranted. Ultimately, in solid non-sclerotic soft tissue or bone lesions the literature supports an average number of 5–6 core samples (16–18 gage) be obtained for the purposes of diagnosis alone, and up to 8–10 core biopsy samples in lesions demonstrating the greatest degrees of heterogeneity. In medullary or cortical bone lesions with varying degrees of sclerosis, hard bone biopsy devices, commonly 11-gage, or larger (Figure 5), provide sizeable specimens in fewer passes (Jelinek et al., 2002; Puri et al., 2006). Communication with the surgeon or interventional radiologist performing these procedures is crucial to assure that adequate tissue is obtained at the time of biopsy and the pathologist should be present during the procedure to assure adequacy of tissue.

It is recognized that the illustrated case represents a rather ideal scenario that will not apply to all cases. Fresh tumor tissue banking will be more challenging in cases where only smaller biopsies are possible, or where the work-up indicates substantial tumor necrosis or extensive intermixed normal tissue invaded by tumor. In such cases, tissue needs for a confident diagnostic work-up obviously supersede those for banking. However, it is likely that at least small amounts of tissue can be banked from many biopsies. We also strongly encourage physicians to submit tissue from metastases when available after biopsy. Although often metastatic sites are not biopsied at the time of diagnosis or relapse, when performed, submission of this tissue for banking is optimal. It cannot be overemphasized how critical such efforts are to advancing our understanding of Ewing sarcoma pathobiology and treatment.

### Summary and conclusions

Over the past 10 years, the COG has collected over 1000 Ewing sarcoma primary tumor specimens that have been banked and distributed to investigators. These tissue samples have been used to advance our understanding of Ewing sarcoma pathogenesis. We anticipate that the next decade will bring additional improvements in treatment through a better understanding of the biology of this devastating disease, especially for older patients with advanced disease at presentation.

## Conflict of Interest Statement

The authors declare that the research was conducted in the absence of any commercial or financial relationships that could be construed as a potential conflict of interest.
